# Robust network data envelopment analysis approach to evaluate the efficiency of regional electricity power networks under uncertainty

**DOI:** 10.1371/journal.pone.0184103

**Published:** 2017-09-27

**Authors:** Mohsen Fathollah Bayati, Seyed Jafar Sadjadi

**Affiliations:** Department of Industrial Engineering, Iran University of Science and Technology, Tehran, Iran; Chongqing University, CHINA

## Abstract

In this paper, new Network Data Envelopment Analysis (NDEA) models are developed to evaluate the efficiency of regional electricity power networks. The primary objective of this paper is to consider perturbation in data and develop new NDEA models based on the adaptation of robust optimization methodology. Furthermore, in this paper, the efficiency of the entire networks of electricity power, involving generation, transmission and distribution stages is measured. While DEA has been widely used to evaluate the efficiency of the components of electricity power networks during the past two decades, there is no study to evaluate the efficiency of the electricity power networks as a whole. The proposed models are applied to evaluate the efficiency of 16 regional electricity power networks in Iran and the effect of data uncertainty is also investigated. The results are compared with the traditional network DEA and parametric SFA methods. Validity and verification of the proposed models are also investigated. The preliminary results indicate that the proposed models were more reliable than the traditional Network DEA model.

## Introduction

Efficiency evaluation of the electricity power networks is an important task for managers for better understanding the past accomplishments of the network, which helps them plan for its future development. In fact, understanding the efficiency level of homogenous electricity power networks may help decision makers and regulators adopt better management strategies and make realistic policies.

Several methods have been developed to evaluate the efficiency of decision making units (DMUs). These methods can be generally classified as parametric and non-parametric methods. While in the parametric methods a cost or production function is estimated, the advantage of non-parametric methods is that it is not necessary to estimate the cost or production function.

Data envelopment analysis (DEA) model, developed by Charnes et al. [1] is a non-parametric methodology which has been widely recognized as an effective technique for measuring and evaluating the relative efficiency of a set of decision making units in the presence of multiple inputs and outputs. DEA applies an efficient frontier made up of the most efficient decision making units to measure the relative efficiency of different decision making units. The basic idea behind DEA method is application of mathematical optimization for evaluating relative efficiency of DMUs with multiple inputs and outputs.

In traditional DEA models, internal structure of DMUs is ignored and each DMU is considered as a black box. However, there are studies that show this assumption may be misleading [2, 3]. In literature, DEA models that consider the internal structure of DMU's are called the Network DEA models.

In many practical applications, data are subject to uncertainty. According to [4], sometimes even small data perturbation yields suboptimal or infeasible solutions. It means that considering certain data in DEA, may lead to misleading efficiency results. Since, a small change in data may change the classification of decision making units (DMUs) from an efficient to an inefficient status and vice versa [5].

In this paper, Robust Network DEA models are developed to evaluate the efficiency of some electricity power networks with uncertain data. Robust optimization is a relatively new methodology for dealing with data uncertainty. While there are many methods to deal with uncertainty in data, an important motivation for applying robust optimization method is that in many applications the probability distribution of the data in unknown. Furthermore, in some applications infeasibility is not allowed at all. The proposed models are based on the adaptation of robust optimization methods developed by Ben-Tal and Nemirovski [4] and Bertsimas et al. [6]. The proposed robust optimization techniques have not been applied with Network DEA in any other previous studies.

This paper is organized into seven sections. Literature review is presented in section 2. Mathematical details of the network DEA model and proposed Robust Network DEA (RNDEA) models are illustrated in sections 3 and 4, respectively. The proposed models are based on Ben-Tal and Nemirovski (BN) and Bertsimas et al. (BA) approaches. Section 5 represents the stochastic frontier analysis (SFA) method. A case study of 16 regional electricity power networks is presented in section 6. Moreover, in this section the results of the implementation of Network DEA, Robust Network DEA based on BN and BA approaches and SFA method are illustrated and compared. Finally, the conclusions are summarized in Section 7.

## Literature review

Mathematical optimization is a branch of applied mathematics that generally tries to optimize a real objective function by selecting best values for decision variables. Mathematical optimization techniques can be applied to improve electricity power systems in different ways (e.g., [7, 8]). Hu et al.[9] applied a mathematical optimization framework to optimize plug-in hybrid electric vehicles. Hu et al. [10] developed a multicriteria optimization approach for evaluating the optimal tradeoffs between the fuel-cell durability and hydrogen economy in the fuel-cell hybrid bus. DEA transforms the problem of efficiency evaluation to a relatively simple linear programming optimization model, in which, the value of objective function is the relative efficiency of under consideration DMU.

In the previous studies, DEA was applied for measuring the relative efficiency of the electricity utilities. Edvardsen and Førsund [11] studied a sample of large electricity distribution utilities from Norway, Sweden, Denmark, Finland and The Netherlands for the year 1997, using input-oriented DEA and Malmquist productivity index. They found that electricity distributors of Finland maintained the highest productivity compared with other countries. Estache et al. [12] used DEA and stochastic frontier analysis to evaluate the efficiency of the main electricity distribution companies in South America. They found a low correlation between DEA and stochastic frontier analysis. Giannakis et al. [13] applied DEA to study service quality of electricity distribution utilities in UK. They found that cost-efficient companies had not necessarily shown high service quality. They also concluded that integrating service quality in regulatory benchmarking was preferable to cost-only approaches. Ramos-Real et al. [14] estimated changes in the productivity of the 18 Brazilian electricity distribution companies using DEA. They found that the incentives generated in the reform process would not have led the companies to behave in a more efficient manner. Sadjadi and Omrani [15] developed a new DEA method with the consideration of uncertainty in the outputs to evaluate the efficiency of 38 electricity distribution companies in Iran. Tavana et al. [16] proposed a DEA model to investigate the impact of IT investment on productivity of 20 Iranian power plants. Sözen et al. [17] applied CRS and VRS models to evaluate the efficiency of power plants in Turkey with respect to the cost of electricity generation and the environmental effects. Furthermore, they investigated the relationship between efficiency scores and input/output factors. Vazhayil and Balasubramanian [18] grouped Indian electricity sector strategies into three portfolios and employed deterministic and stochastic DEA models for efficiency optimization of electricity sector strategies. Their analysis showed that weight-restricted stochastic DEA model was more appropriate than deterministic method.

Existing DEA approaches for evaluating the efficiency of electricity utilities are under some serious criticisms: In large body of literature, only one stage (component) of electricity network is evaluated. For example power plants [16, 17] or electricity distribution firms [11–15]. Furthermore, in most of the existing approaches, data uncertainty is ignored. Data uncertainty is considered in robust DEA model proposed by Sadjadi and Omrani [15] to evaluate the efficiency of stage distribution, but in this study only outputs were considered to be uncertain and uncertainty in inputs was ignored. In this paper, network DEA is applied for evaluating the efficiency of entire electricity networks and robust optimization methodology is used for dealing with data uncertainty.

The idea of network DEA was first developed by Charnes et al. [19]. They discussed two processes of army recruitment: a) awareness creation through advertising and b) contract creation. Since then, several studies have been accomplished to measure the efficiency of systems taking into account the internal structures. Färe et al. [20] introduced the basic network DEA models to investigate the efficiency of sub DMUs. Prieto and Zofío [21] developed a network DEA model to deal with different sub-technologies corresponding to alternative production processes, to evaluate the efficient resource allocation among them. They applied their model to a set of OECD countries (OECD -The Organization for Economic Co-operation and Development- is founded in 1960 to promote policies that will improve the economic and social well-being of people around the world. Today, 35 countries are members of OECD). Cook et al. [22] proposed several network DEA models to examine a more general problem of an open multistage process. Kao [23] developed a relational network DEA model by considering interrelationship of the sub-DMUs, to measure the efficiency of the whole system and sub-systems at the same time. They also introduced dummy processes to transform a complicated system to a series system. The model evaluated both overall efficiency and divisional efficiency. Saranga and Moser [24] applied an external assessment survey methodology that complements the internal perceptional measures of purchasing, supplied management (PSM) performance and developed an efficiency measurement framework using the classical and two-stage value chain data envelopment analysis models. Yu et al. [25] designed information-sharing scenarios to analyze the efficiency of supply chain through a simulation model. They applied a cross-efficiency DEA approach to deal with both desirable and undesirable measures. Chen and Yan [26] took the perspective of organization mechanism to deal with the complex interactions in supply chain. Accordingly, they introduced three network DEA models, under the concepts of centralized, decentralized and mix organization mechanisms. A comprehensive review of the network DEA models was accomplished by Kao [27]. He classified different network DEA models with regard to both the models developed and structures examined.

In DEA literature, stochastic approach [18], interval model [28, 29] and fuzzy method [30, 31] are also applied to model data uncertainty. One of drawbacks of stochastic approach is that the decision maker is required to assume a distribution function for the error process [32]. However this assumption may not be realistic because it is very difficult to choose one distribution over another. The interval approach was first proposed by Cooper et al. [33]. One of difficulties of this approach is difficulty in the evaluation of the upper and lower bounds of the relative efficiencies of the DMUs. The fuzzy DEA was first proposed by Sengupta [32]. In some cases the complexity of fuzzy approach can grow exponentially. Pitfalls of some fuzzy DEA models are addressed by Soleimani-Damaneh et al. [34]. Because of these drawbacks of existing methods for dealing with data uncertainty, robust optimization method is applied in this paper. Robust optimization was originally presented by Soyster [35]. El-Ghaoui and Lebret [36] and El Ghaoui et al. [37] and Ben-Tal and Nemirovski [4, 38, 39] presented a new idea for dealing with the data uncertainty based on ellipsoidal uncertainty sets. Recently, Bertsimas and Sim [40–42] and Bertsimas et al. [6] introduced a robust optimization approach based on polyhedral uncertainty set. Robust optimization theory has been applied in many practical applications. Such examples include project management (e.g., [43–45]), inventory management (e.g., [46, 47]), portfolio optimization (e.g., [48–50]), environmental management (e.g., [51, 52]). Comprehensive information about the history and the growth of the robust optimization can be found at [53] and [54].

## Network data envelopment analysis

To evaluate the efficiency of electricity power networks, it is not adequate to only consider the initial inputs and final outputs of networks and ignore the internal linking activities among different stages, because ignoring the operations of components may lead us to misleading results. More significantly, a network may be efficient while all components are not [55].

**Fig 1:**
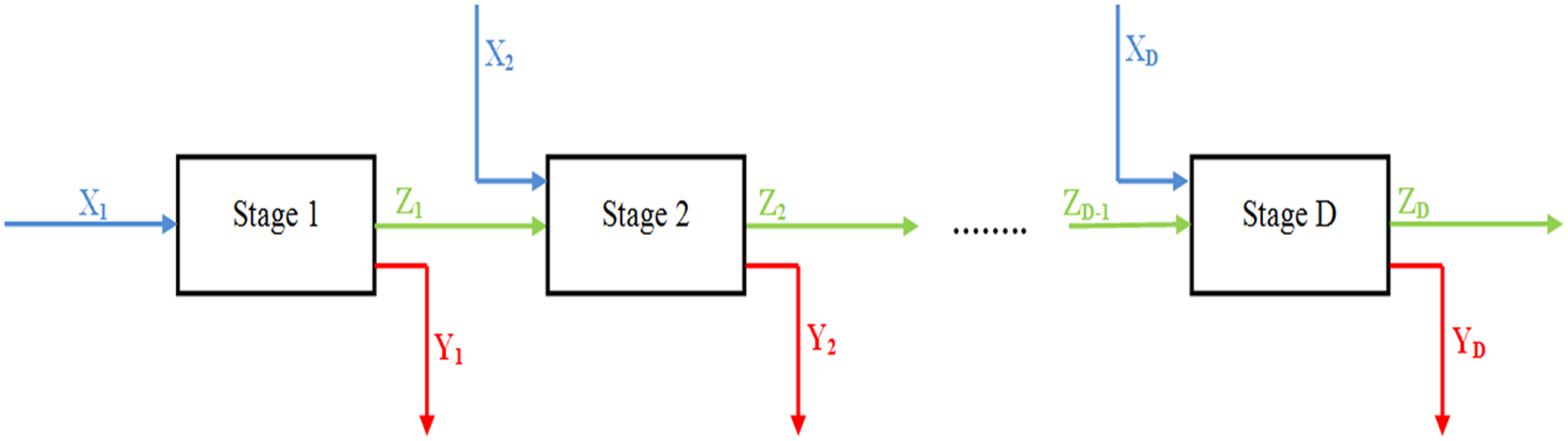
General structure of the D-stage serial process [22].

Consider the *D*-stage process pictured in Fig 1. The input vector of each stage is denoted by *X*_*d*_ (*d = 1*, …, *D*). The outputs of stage *d (d = 1*, …, *D*) take two forms, *Y*_*d*_ and *Z*_*d*_. *Y*_*d*_ represents outputs leaving the process at stage *d* and *Z*_*d*_ represents the outputs of stage *d* that becomes inputs to the next stage, *d+1*. These types of outputs are intermediate measures.

Linear programming form of the network DEA model was proposed by Cook et al. [22] to evaluate the efficiency of the general serial structures presented in model (1). In this model the efficiency of DMU under consideration (DMU O) is *π*_*O*_, and *u*_*dr*_, *v*_*di*_ and *w*_*dk*_ are the input, output and intermediate measures weights assigned to *r*th output, *i*th input and *k*th intermediate measure of stage *d*. *x*_*dij*_ (*d = 1*, …, *D*, *i = 1*, …, *I*, *j = 1*, …, *n*) and *y*_*drj*_ (*d = 1*, …, *D*, *r = 1*, …, *R*, *j = 1*, …, *n*) are the *i*th input and *r*th output of stage *d* in DMU *j*. *z*_*dkj*_ denotes *k*th outputs of stage *d* in DMU *j* that becomes the inputs to the stage *d+1*.

$$\begin{array}{l} \begin{array}{ll} \text{max}\;\pi_{O}= & \sum_{d=1}^{D}\sum_{r}u_{dr}y_{drO}+\sum_{d=1}^{D-1}\left(\sum_{k}w_{dk}z_{dkO}\right)\\ S.T: & \\ & \sum_{d}\left(\sum_{i}v_{di}x_{diO}\right)+\sum_{d>1}\left(\sum_{k}w_{d-1,k}z_{d-1kO}\right)=1\;\\ & \sum_{k}w_{1k}z_{1kj}+\sum_{r}u_{1r}y_{1rj}\leq \sum_{i}v_{1i}x_{1ij}\\ & \sum_{k}w_{dk}z_{dkj}+\sum_{r}u_{dr}y_{drj}\leq \sum_{i}v_{di}x_{dij}+\sum_{k}w_{d-1,k}z_{d-1kj}\;\;\;\forall j,d>1 \end{array}\\ v_{di},u_{dr},w_{dk}>0 \end{array}$$ (1)

Model (1) is an output-oriented network DEA model. A DEA model is output-oriented if it maximizes outputs without increasing inputs. In this model, the objective function is maximizing the summation of outputs of different stages of DMU O. First constraint keeps the level of inputs at a constant level. Constraint 2 and constraint set 3 state that the aggregated system output must be less than or equal tothe aggregated system input for all DMUs. Constraint 2 refers to stage 1 and constraint set 3 refers to other stages.

## Robust optimization

Robust optimization provides risk-averse models to deal with uncertainty in optimization problems. In traditional optimization methodologies, data are assumed to be known with certainty. In fact in traditional methods a small data uncertainty is ignored hoping that small data uncertainties would not have significant impact on optimality and feasibility of the solution, but as illustrated by Ben-Tal and Nemirovski [4], sometimes even a small data perturbation deserves suboptimal or infeasible solutions.

In order to present the robust methods proposed by Ben-Tal and Nemirovski [4] and Bertsimas et al. [6], consider the following linear optimization problem:

$$\begin{array}{ll} \text{min}\;c^{\prime }x & \\ subject\;to: & \\ & Ax\geq b,\\ & x\in X \end{array}$$ (2)

Let *J*_*i*_ be the set of uncertain coefficients in *i*^*th*^ row of matrix *A* and ${\tilde{a}}_{ij}$(*j* ∈ *J*_*i*_) be the true values of the parameters which are subject to uncertainty and take value in [$a_{ij}-{\hat{a}}_{ij},a_{ij}+{\hat{a}}_{ij}$], where *a*_*ij*_ and ${\hat{a}}_{ij}$ are the nominal value and the constant perturbation of variable ${\tilde{a}}_{ij}$, respectively.

Since the equality constraints are not allowed in robust optimization methods, model (1) is transformed into the following form, which can be solved easily using parametric programming method.

$$\begin{array}{ll} \text{max}\;\pi_{O} & \\ S.T: & \\ & \sum_{d=1}^{D}\sum_{r}u_{dr}y_{drO}+\sum_{d=1}^{D-1}\left(\sum_{k}w_{dk}z_{dkO}\right)-\pi_{O}\sum_{d}\left(\sum_{i}v_{di}x_{diO}\right)-\pi_{O}\sum_{d>1}\left(\sum_{k}w_{d-1,k}z_{d-1kO}\right)\geq 0\\ & \sum_{k}w_{1k}z_{1kj}+\sum_{r}u_{1r}y_{1rj}\leq \sum_{i}v_{1i}x_{1ij}\\ & \sum_{k}w_{dk}z_{dkj}+\sum_{r}u_{dr}y_{drj}\leq \sum_{i}v_{di}x_{dij}+\sum_{k}w_{d-1,k}z_{d-1kj}\;\;\;\forall j,d>1\\ v_{di},u_{dr},w_{dk} & >0 \end{array}$$ (3)

In model (3), the *π*_*O*_ is overall efficiency of DMU O. The first constraint is transformation of output/input ratio ($\left(\sum_{d=1}^{D}\sum_{r}u_{dr}y_{drO}+\sum_{d=1}^{D-1}\left(\sum_{k}w_{dk}z_{dkO}\right)\right)/\left(\sum_{d}\left(\sum_{i}v_{di}x_{diO}\right)+\sum_{d>1}\left(\sum_{k}w_{d-1,k}z_{d-1kO}\right)\right)\geq \pi_{O}$). In this model, when we maximize *π*_*O*_, the output/input ratio of DMU O (efficiency of DMU O) will be calculated. Same as model (1), other constraints state that the aggregated system output must be less than or equal to the aggregated system input for all DMUs.

### Robust network DEA based on BN approach

Ben-Tal and Nemirovski define the uncertain data ${\tilde{a}}_{ij}$ as follows [4]:

$${\tilde{a}}_{ij}=(1+e\zeta_{ij})a_{ij}\;\;\forall j\in J_{i}$$ (4)

where *ζ*_*ij*_ represent independent random variables which are symmetrically distributed between -1 and 1 and *e>0* is the percentage of perturbations (uncertainty level). Robust form of model (2) based on BN approach is as follows:

$$\begin{array}{ll} \text{min}\;c^{\prime }x & \\ subject\;to: & \\ & \sum_{j}a_{ij}x_{j}-e\left[\sum_{j\in J_{i}}\left|a_{ij}\right|y_{ij}+\Omega \sqrt{\sum_{j\in J_{i}}a_{ij}^{2}z_{ij}^{2}}\right]\geq b_{i},\;\;\forall i\\ & -y_{ij}\leq x_{j}-z_{ij}\leq y_{ij}\;\;\forall i,j \end{array}$$ (5)

In model (5), robust form of general linear programming problems based on BN approach is presented. This model shows that BN approach adds a term to the left side of the constraints.

In this approach decision maker can control the reliability level by varying parameter Ω.

The proposed robust network DEA based on BN approach is expressed as follows. This model is a general form that considers inputs, outputs and intermediate measures as uncertain data.

$$\begin{array}{ll} \text{max}\;\pi_{O} & \\ S.T: & \\ & \begin{array}{ll} \sum_{d=1}^{D}\sum_{r}u_{dr}y_{drO} & +\sum_{d=1}^{D-1}\left(\sum_{k}w_{dk}z_{dkO}\right)-\pi_{O}\sum_{d}\left(\sum_{i}v_{di}x_{diO}\right)-\pi_{O}\sum_{d>1}\left(\sum_{k}w_{d-1,k}z_{d-1kO}\right)\\ & -e\left[\sum_{d}^{D}\sum_{r}^{R_{d}}\left|y_{dro}\right|Y_{dro}+\sum_{d}^{D}\sum_{i}^{I_{d}}\left|x_{dio}\right|X_{dio}+\sum_{d}^{D}\sum_{k}^{K_{d}}\left|z_{dko}\right|Z_{dko}\right]\\ & -e\left[\Omega \sqrt{\sum_{d}^{D}\sum_{r}^{R_{d}}{\left(y_{dro}^{}W_{dro}^{y}\right)}^{2}+\sum_{d}^{D}\sum_{i}^{I_{d}}{\left(x_{dio}^{}W_{dio}^{x}\right)}^{2}+\sum_{d}^{D}\sum_{k}^{K_{d}}(z_{dko}^{}W_{dko}^{z}}{)}^{2}\right]\geq 0 \end{array}\\ & \begin{array}{ll} \sum_{k}w_{1k}z_{1kj} & +\sum_{r}u_{1r}y_{1rj}-\sum_{i}v_{1i}x_{1ij}+e\left[\sum_{r}^{R_{1}}\left|y_{1ro}\right|Y_{dro}+\sum_{i}^{I_{1}}\left|x_{1io}\right|X_{1io}+\sum_{k}^{K_{1}}\left|z_{1ko}\right|Z_{1ko}\right]\\ & +e\left[\Omega \sqrt{\sum_{r}^{R_{1}}(y_{1rj}^{}W_{1rj}^{y}{)}^{2}+\sum_{i}^{I_{1}}(x_{1ij}^{}W_{1ij}^{x}{)}^{2}+\sum_{k}^{K_{1}}{\left(z_{1kj}^{}W_{1kj}^{z}\right)}^{2}}\right]\leq 0 \end{array}\\ & \begin{array}{ll} \sum_{k}w_{dk}z_{dkj} & +\sum_{r}u_{dr}y_{drj}-\sum_{i}v_{di}x_{dij}-\sum_{k}w_{d-1,k}z_{d-1kj}+e\left[\sum_{d}^{D}\sum_{r}^{R_{d}}\left|y_{drj}\right|Y_{drj}+\sum_{d}^{D}\sum_{i}^{I_{d}}\left|x_{dij}\right|X_{dij}+\sum_{d}^{D}\sum_{k}^{K_{d}}\left|z_{dkj}\right|Z_{dkj}\right]\\ & +e\left[\Omega \sqrt{\sum_{d}^{D}\sum_{r}^{R_{d}}{\left(y_{drj}^{}W_{drj}^{y}\right)}^{2}+\sum_{d}^{D}\sum_{i}^{I_{d}}{\left(x_{dij}^{}W_{dij}^{x}\right)}^{2}+\sum_{d}^{D}\sum_{k}^{K_{d}}{\left(z_{dkj}^{}W_{dkj}^{z}\right)}^{2}}\right]\leq 0\;\;\;\forall j,d>1 \end{array}\\ & -Y_{drj}\leq u_{dr}-W_{drj}^{y}\leq Y_{drj}\;\;\;\forall d,r,j\\ & -X_{dij}\leq v_{di}-W_{dij}^{x}\leq X_{dij}\;\;\;\forall d,i,j\\ & -Z_{dkj}\leq w_{dk}-W_{dkj}^{z}\leq Z_{dkj}\;\;\;\forall d,k,j\\ v_{di},u_{dr},w_{dk} & >0\;\;\;\forall i,r,k,j \end{array}$$ (6)

In model (6), *π*_*O*_ is the efficiency of under consideration DMU and *x*_*dij*_, *y*_*drj*_ and *z*_*dkj*_ are *i*^*th*^ input, *r*^*th*^ output and *k*^*th*^ intermediate measure of division *d* in *j*^*th*^ DMU, respectively. Constraints of model (6), are robust form of constraints of model (3) based on BN approach (see model (5)). This model is in nonlinear form and can be solved using nonlinear programming techniques.

Unfortunately the BN approach suffers from the following disadvantages:

- The approach increases the number of variables and makes the models more complicated.
- BN approach transfers linear programming models into nonlinear forms which are more difficult to obtain optimal solutions.

### Robust network DEA based on BA approach

Let *λ*_*ij*_ be the scaled deviation of parameter ${\tilde{a}}_{ij}$ from its nominal value as $\lambda_{ij}=({\tilde{a}}_{ij}-a_{ij})/{\hat{a}}_{ij}$. Clearly, *η*_*ij*_ is unknown and symmetrically distributed in the interval [–1,1]. In addition, *J*_*i*_ is the set of coefficients in constraint *i* which are uncertain. Moreover, the parameter Γ_*i*_ is called budget of uncertainty and introduced for constraint *i*, to adjust level of protection against uncertainty. The summation of scaled variation of the parameters cannot exceed Γ_*i*_, i.e., $\sum_{j=1}^{n}\lambda_{ij}\leq \Gamma_{i}$. This parameter takes values in [0,|*J*_*i*_|], by taking Γ_*i*_ = 0 and Γ_*i*_ = |*J*_*i*_| the problem obtains its nominal and the worst case, respectively. If the thresholds Γ_*i*_ takes the values in the interval (0,|*J*_*i*_|), the decision maker makes a trade-off between robustness and performance.

Let ${\tilde{a}}_{i}$ be the *i*^*th*^ vector of *A*′. Problem (2) can be reformulated as follow:

$$\begin{array}{ll} \text{min}\;c^{\prime }x & \\ subject\;to: & \\ & {\tilde{a^{\prime }}}_{i}x\geq b_{i},\;\forall i,{\tilde{a}}_{i}\in J_{i}\\ & x\in X \end{array}$$ (7)

In model (7), the *i*th constraint is equivalent to ${\text{min}}_{{\tilde{a}}_{i}\in J_{i}}{\tilde{a^{\prime }}}_{i}x\geq b_{i}$. As a result, for constraint *i*, the following auxiliary problem has to be solved:

$$\begin{array}{ll} -\text{max}\;\sum_{j=1}^{n}{\hat{a}}_{ij}\left|x_{j}\right|\lambda_{ij} & \\ subject\;to: & \\ & \sum_{j=1}^{n}\lambda_{ij}\leq \Gamma_{i}\;\;\forall i\\ & 0\leq \lambda_{ij}\leq 1\;\;\forall i \end{array}$$ (8)

Dual form of Eq (8) is as follow:

$$\begin{array}{ll} \text{min}\;\Gamma_{i}q_{i}+\sum_{j\in J_{i}}r_{ij} & \\ subject\;to: & \\ & q_{i}+r_{ij}\geq ea_{ij}\left|x_{j}^{*}\right|\;\;\forall i,j\in J_{i}\\ & r_{ij}\geq 0\;\;\forall j\in J_{i}\\ & q_{i}\geq 0\;\;\forall i \end{array}$$ (9)

where *q*_*i*_ and *r*_*ij*_ are the dual variables. Substituting Eq (9) in Eq (7), the robust approach based on [40–42] and [6] is as follow:

$$\begin{array}{ll} \text{min}\;c^{\prime }x & \\ subject\;to: & \\ & {a^{\prime }}_{i}x-\Gamma_{i}q_{i}-\sum_{j\in J_{i}}r_{ij}\geq 0\;\;\forall i,\\ & q_{i}+r_{ij}\geq ea_{ij}y_{j}\;\;\forall i,j,\\ & -y_{j}\leq x_{j}\leq y_{j}\;\;\forall j,\\ & q_{i},r_{ij}\geq 0\;\;\forall i\\ & x\in X \end{array}$$ (10)

The proposed robust network DEA based on BA approach is expressed in Eq (11).

$$\begin{array}{ll} \text{max}\;Z_{O}=\pi & \\ S.T: & \\ & \begin{array}{ll} \sum_{d=1}^{D}\sum_{r}u_{dr}y_{drO} & +\sum_{d=1}^{D-1}\left(\sum_{k}w_{dk}z_{dkO}\right)-\pi \sum_{d}\left(\sum_{i}v_{di}x_{diO}\right)-\pi \sum_{d>1}\left(\sum_{k}w_{d-1,k}z_{d-1kO}\right)\\ & -\sum_{d=1}^{D}q_{d}^{y}\Gamma_{d}^{y}-\sum_{d}^{D}\sum_{r=1}^{R_{d}}r_{dro}^{y}-\sum_{d=1}^{D}q_{d}^{x}\Gamma_{d}^{x}-\sum_{d}^{D}\sum_{i=1}^{I_{d}}r_{dio}^{x}-\sum_{d=1}^{D-1}q_{d}^{z}\Gamma_{d}^{z}-\sum_{d}^{D}\sum_{k=1}^{K_{d}}r_{dko}^{z}\geq 0 \end{array}\\ & \begin{array}{ll} \sum_{k}w_{1k}z_{1kj} & +\sum_{r}u_{1r}y_{1rj}-\sum_{i}v_{1i}x_{1ij}+q_{1}^{y}\Gamma_{1}^{y}+\sum_{r=1}^{R_{1}}r_{1rj}^{y}+q_{1}^{x}\Gamma_{1}^{x}+\sum_{i=1}^{I_{1}}r_{1ij}^{x}+q_{1}^{z}\Gamma_{1}^{z}+\sum_{k=1}^{K_{1}}r_{1kj}^{z}\leq 0\\ \sum_{k}w_{dk}z_{dkj} & +\sum_{r}u_{dr}y_{drj}-\sum_{i}v_{di}x_{dij}-\sum_{k}w_{d-1,k}z_{d-1kj}+q_{d}^{y}\Gamma_{d}^{y}+\sum_{d}^{D}\sum_{r=1}^{R_{d}}r_{drj}^{y}+q_{d}^{x}\Gamma_{d}^{x}+\sum_{d}^{D}\sum_{i=1}^{I_{d}}r_{dij}^{x}\\ & +q_{d-1}^{z}\Gamma_{d-1}^{z}+\sum_{d}^{D}\sum_{k=1}^{K_{d}}r_{dkj}^{z}\leq 0\;\;\;\forall j,d>1 \end{array}\\ & q_{d}^{x}+r_{dij}^{x}\geq e^{x}x_{dij}t_{di}^{x}\;\;\;\forall d,i,j\\ & q_{d}^{y}+r_{drj}^{y}\geq e^{y}y_{drj}t_{dr}^{y}\;\;\;\forall d,r,j\\ & q_{d}^{z}+r_{dkj}^{z}\geq e^{z}z_{dkj}t_{dk}^{z}\;\;\;\forall d,k,j\\ & \begin{array}{l} -t_{di}^{x}\leq v_{di}\leq t_{di}^{x}\;\;\;\forall d,i\\ -t_{dr}^{y}\leq u_{dr}\leq t_{dr}^{y}\;\;\;\forall d,r\\ -t_{dk}^{z}\leq w_{dk}\leq t_{dk}^{z}\;\;\;\forall d,k \end{array}\\ v_{di},u_{dr},w_{dk},q_{d}^{x},q_{d}^{y},q_{d}^{z},r_{ij}^{x},r_{rj}^{y},r_{kj}^{z} & >0\;\;\;\forall i,r,k,d,j \end{array}$$ (11)

Obviously, constraints of model (11) are robust form of constraints of model (3) based on BA approach. In fact, model (11) is developed form of model (3) based on model (10).

## Stochastic frontier analysis (SFA)

Stochastic Frontier Analysis (SFA), which was independently proposed by Aigner et al. [56] and Meeusen and Van den Broeck [57] is a statistical method based on the regression analysis for estimating the efficient frontier and efficiency scores [58]. Statistical nature of SFA allows for the inclusion of stochastic errors in the analysis. SFA decomposes the error term in two parts, one represents the statistical noise and another represents the inefficiency.

The stochastic frontier production function model presented by Aigner et al. [56] and Meeusen and Van den Broeck [57] is in following form:

$$\text{ln}(q_{i})=x_{i}\beta +v_{i}-u_{i}$$ (12)

where *x*_*i*_, *q*_*i*_, and *e*_*i*_ = *v*_*i*_ − *u*_*i*_ are input vector, output vector and error term for *DMU*_*i*_, respectively. *β*_*i*_ is the vector of unknown parameters that should be estimated and *v*_*i*_ is the symmetric error term and *u*_*i*_ is asymmetric non-negative inefficiency term. Kumbhakar and Lovell [59] defined the relationship between technical efficiency and −*u*_*i*_ as: *TE*_*i*_ = exp(−*u*_*i*_) where *TE*_*i*_ is the technical efficiency of *DMU*_*i*_. In order to compute the technical efficiency of decision making units, the probability function for the distribution of the errors and distribution of inefficiencies is required. The probability distributions function for *v*_*i*_ and *u*_*i*_ are normally assumed as follows:

$$v_{i}\text{\textasciitilde{}N(0,}\sigma_{\text{v}}^{\text{2}})$$ (13)

$$u_{i}{\text{\textasciitilde{}N}}^{+}\text{(}\mu \text{,}\sigma_{\text{u}}^{\text{2}})$$ (14)

where $\text{N(0,}\sigma_{\text{v}}^{\text{2}})$ and ${\text{N}}^{+}\text{(}\mu \text{,}\sigma_{\text{u}}^{\text{2}})$ in Eqs (13) and (14)represent that distribution functions of *v*_*i*_ and *u*_*i*_ are normal and half-normal [59]. Also, *γ* is defined as follow:

$$\gamma =\frac{\sigma_{u}^{2}}{\sigma_{u}^{2}+\sigma_{v}^{2}}$$ (15)

In Eq (15), the parameter *γ* is the relative importance of inefficiency. This parameter must be between 0 and 1 and shows the percentage of error that the efficiency may have [59]. In models with single output, functions such as Cobb-Douglas are applied to estimate the efficiency but in multi-input and multi-output state, distance function is required. As in [60] and [61], for distance function the translog form is applied. Translog form of input distance function is as below:

$$\begin{array}{ll} \text{ln}(D_{Ii}/x_{Ki}) & =\alpha_{0}+\sum_{m=1}^{M}\alpha_{m}\text{ln}y_{mi}+\frac{1}{2}\sum_{m=1}^{M}\sum_{n=1}^{M}\alpha_{mn}\text{ln}y_{mi}\text{ln}y_{ni}\\ & +\sum_{k=1}^{K-1}\beta_{k}\text{ln}x_{ki}^{*}+\frac{1}{2}\sum_{k=1}^{K-1}\sum_{l=1}^{K-1}\beta_{kl}\text{ln}x_{ki}^{*}\text{ln}x_{li}^{*}+\sum_{k=1}^{K-1}\sum_{m=1}^{M}\delta_{km}\text{ln}x_{ki}^{*}\text{ln}y_{mi}\;\;\;i=1,\ldots ,n \end{array}$$ (16)

where *i* denotes *i*^*th*^ decision making unit and M and K are the number of inputs and outputs and $x_{k}^{*}=x_{k}/x_{K}$. Eq (16) may be more clearly expressed as ln(*D*_*Ii*_ /*x*_*Mi*_) = *TL*(*x*_*i*_/*x*_*K*_, *y*_*i*_, *α*, *β*, *δ*), *i = 1*, …, *n*. Eq (16) is re-written as ln(*x*_*Ki*_) = *TL*(*x*_*i*_/*x*_*K*_, *y*_*i*_, *α*, *β*, *δ*) −ln(*D*_*Ii*_), *i = 1*, …, *n*. The −ln(*D*_*Ii*_)is re-expressed as *v*_*i*_−*u*_*i*_. Where *v*_*i*_ is the symmetric error term and *u*_*i*_ is asymmetric non-negative inefficiency term.

## Case study

In this section, the proposed models are applied to evaluate the efficiency of 16 Iranian regional electricity power networks. Iranian regional electricity power networks consist of three stages: Generation, Transmission and Distribution. Each stage has inputs and outputs and specific energy is transmitted between stages. In generation stage, power plants consume gas oil and fuel oil (liquid fuel) and natural gas (gas fuel) to produce electricity power. Generation has two outputs: mean practical power and specific energy. Practical power is maximum power of generators considering environmental situation (temperature, humidity, etc.) and specific energy is total energy produced excluding the electrical energy consumed in power plants. Transmission stage includes stations, overhead lines, cables and other electrical equipment to transit energy from power plants. A station includes a series of electrical equipment e.g. transformers, circuit breakers, disconnectors, instrument devices, in/out-coming feeders etc. number of employees, capacity of transmission stations, length of transmission network and energy delivered from nearby networks are inputs of transmission stage. This stage delivers energy to nearby networks and distribution stage. Stage 3 is distribution stage. This stage consists of a series of medium and low voltage overhead lines and underground cables to distribute electricity energy in an area. Inputs of this stage are: number of employees, length of distribution network and transformers capacity. Transformer is a static electrical device that transfers energy by inductive coupling between its winding circuits. Number of customers and total energy sales are outputs of this stage.

**Fig 2:**
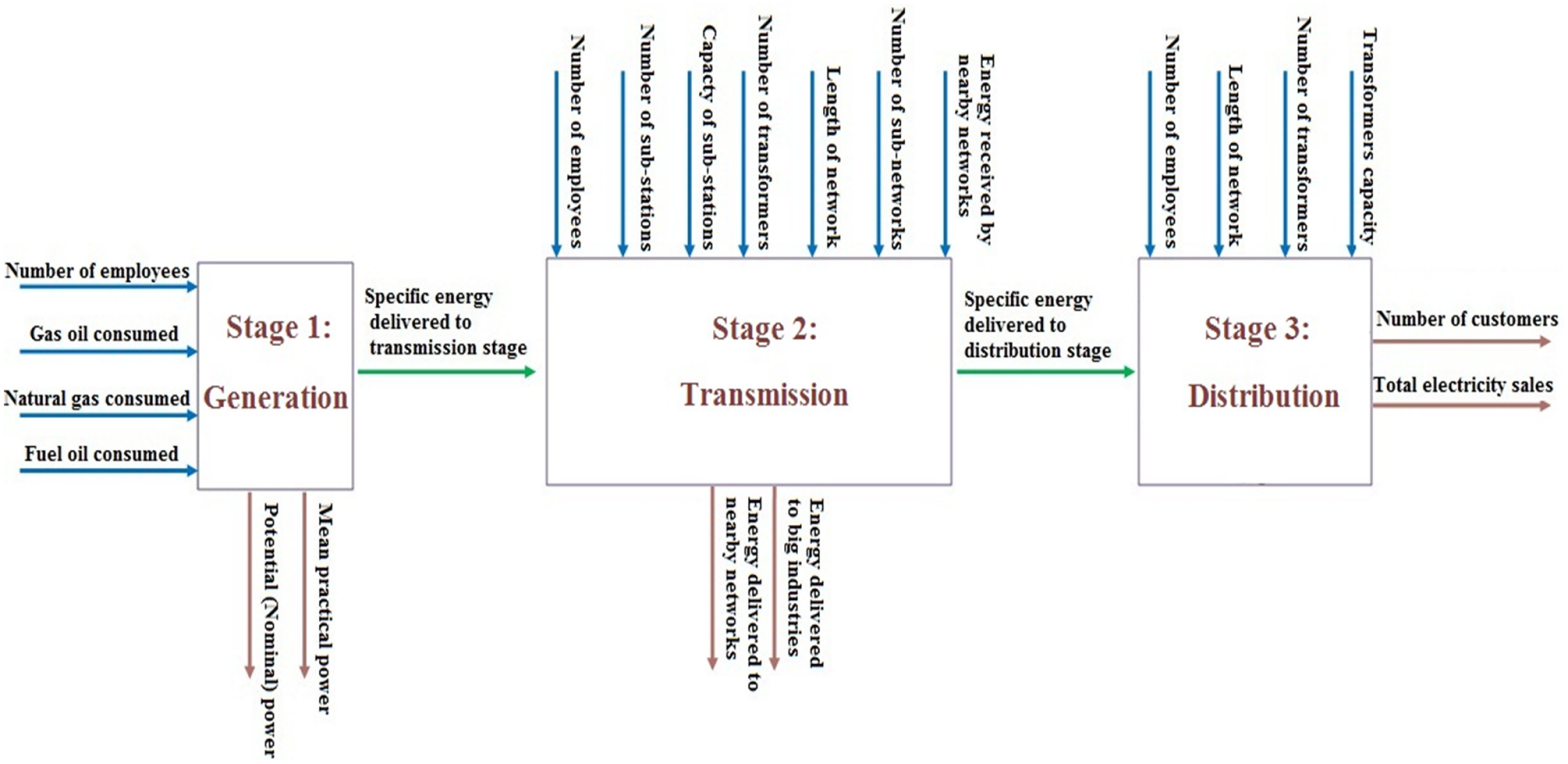
Structure of electricity power networks and considered data.

The regional networks act under the supervision of TAVANIR(Iran power Generation, Transmission and Distribution Management Company). TAVANIR is responsible for managing regional electricity power networks and acts under the supervision of Ministry of Energy. Our data series involves the annual data on 16 regional networks observed in 2014. These data are retrieved from Iran Power Generation, Transmission and Distribution Management Company annual publications. Structure of regional electricity power networks and considered inputs, outputs and intermediate measures for each stage is illustrated in Fig 2.

Summary statistics over data set of case study is shown in Table 1 (to obtain full data see S1 File).

**Table 1:** Summary statistics over data set of case study.

Stage			Max	Min	Mean
Generation	Inputs	Number of employees	1950	60	635
		Liquid fuel consumed (KL)	3660898	85074	1189755.54
		Gas fuel consumed (KM^3^)	9017896	65547	3127147.10
	Outputs	Mean practical power (MW)	13381677	525348	3990465
	Intermediate	Specific energy (MWh)	48880881	2276268	16588837.19
Transmission	Inputs	Number of employees	2358	345	1143
		Capacity of transmission stations (MVA)	52893.5	5423	20135.06
		Length of transmission network (Km)	14529.04	2211.13	7806.59
		Energy received from nearby networks (MWh)	19907000	483284.2	5691148.8
	Outputs	Energy delivered to nearby networks (MWh)	57388000	82579	8433775.98
	Intermediate	Specific energy (MWh)	42640000	2530000	13475666.67
Distribution	Inputs	Number of employees	3061	183	1007.75
		Length of distribution network (Km)	80962.8	11051.4	45831.25
		Transformers capacity (MVA)	22412.4	1241.4	6551.65
	Outputs	Number of customers (*1000)	7877.45	339.13	1977.35
		Total electricity sales (MWh)	37534110	2353407	11245893.88

### Proposed network DEA models results

Table 2 reveals the results of applying Network DEA and Robust Network DEA models for 16 regional electricity power networks (DMUs). As shown in column four of this table, in term of technical efficiency for Network DEA in 2014, five regional networks obtained efficiency score equal to one. These networks can be considered as reference set to the others. Other networks obtained efficiency scores between 0.803 (Kerman) and 0.999 (Tehran, Zanjan and Gilan). Using Network DEA model without considering perturbation in data, the mean overall efficiency of networks is 0.959.

**Table 2:** The results of different approaches.

DMU no	Region	SFA	Network DEA	RNDEA-BN approach			RNDEA-BA approach		
				*e = 0*.*01*	*e = 0*.*05*	*e = 0*.*1*	*e = 0*.*01*	*e = 0*.*05*	*e = 0*.*1*
1	Azarbayejan	0.996	1.000	0.993	0.967	0.934	0.960	0.813	0.699
2	Esfahan	0.929	0.998	0.996	0.976	0.950	0.960	0.810	0.691
3	Bakhtar	0.877	0.836	0.831	0.811	0.787	0.805	0.686	0.576
4	Tehran	0.975	0.999	0.995	0.976	0.951	0.968	0.845	0.754
5	Khorasan	0.962	1.000	0.995	0.973	0.946	0.965	0.829	0.716
6	Khuzestan	0.923	1.000	0.996	0.976	0.928	0.960	0.814	0.711
7	Zanjan	0.964	0.999	0.994	0.969	0.941	0.962	0.818	0.709
8	Semnan	0.923	0.884	0.878	0.855	0.825	0.848	0.710	0.600
9	Sistanvabaluchestan	0.986	1.000	0.996	0.974	0.94	0.960	0.810	0.698
10	Gharb	0.867	0.916	0.909	0.880	0.665	0.880	0.738	0.579
11	Fars	0.776	0.998	0.984	0.974	0.903	0.960	0.810	0.675
12	Kerman	0.943	0.803	0.792	0.777	0.665	0.773	0.654	0.543
13	Gilan	0.924	0.999	0.994	0.973	0.945	0.964	0.827	0.703
14	Mazandaran	0.976	0.998	0.996	0.975	0.957	0.970	0.855	0.753
15	Hormozgan	0.838	0.919	0.914	0.882	0.846	0.884	0.742	0.612
16	Yazd	0.971	1.000	0.993	0.961	0.923	0.960	0.810	0.705
Mean		0.927	0.959	0.954	0.931	0.898	0.924	0.786	0.670
Standard deviation		0.060	0.066	0.067	0.067	0.076	0.065	0.060	0.066

**Fig 3:**
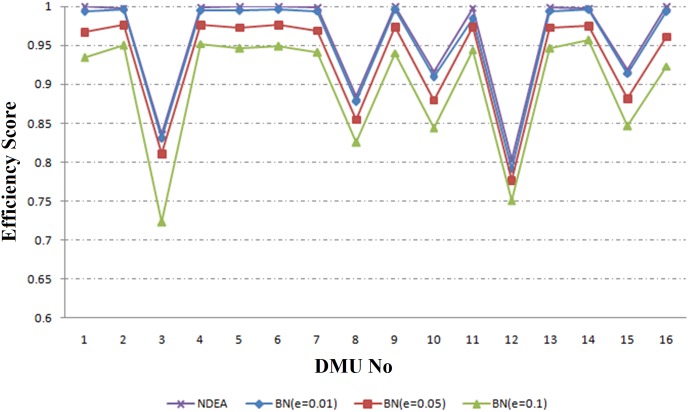
The results of RNDEA-BN approach.

The results of the proposed Robust Network DEA based on BN approach (RNDEA-BN) for reliability level of *κ* = 0.95 (Ω = 0.32) are presented in columns 5–7 of Table 2. The perturbation *e* is considered to be 0.01, 0.05 and 0.1. For example when *e = 0*.*05*, the efficiency scores are varying from 0.777 (Kerman) to 0.976 (Esfahan, Tehran, Khuzestan). The results of RNDEA-BN method for different perturbations are illustrated in Fig 3. In this case as perturbation increases from 0.01 to 0.1, the mean of efficiency measures is decreased from 0.954 to 0.898. In fact, the efficiency score of each network decreases when perturbation increases.

For the implementation of Robust Network DEA based on BA approach (RNDEA-BA) it is sufficient to choose Γ at least equal to Eq (17):

$$\Gamma =1+\Phi^{-1}(1-e)\sqrt{n}$$ (17)

Where Φ is cumulative distribution of the standard Gaussian variable, *e* is perturbation and *n* is the sources of uncertainty. When the problem has few number of uncertain data, it is necessary to ensure full protection [41]. As in this case, each stage has few numbers of inputs, outputs and intermediate measures; it is assumed that $\Gamma_{d}^{x},\;\Gamma_{d}^{y},\Gamma_{d}^{z}$ are equal to the number of inputs, outputs and intermediate measures for each stage respectively. Also, the perturbations are considered to be equal for inputs, outputs and intermediate measures (*e* = *e*^*x*^ = *e*^*y*^ = *e*^*z*^) and are set to 0.01, 0.05 and 0.1.

**Fig 4:**
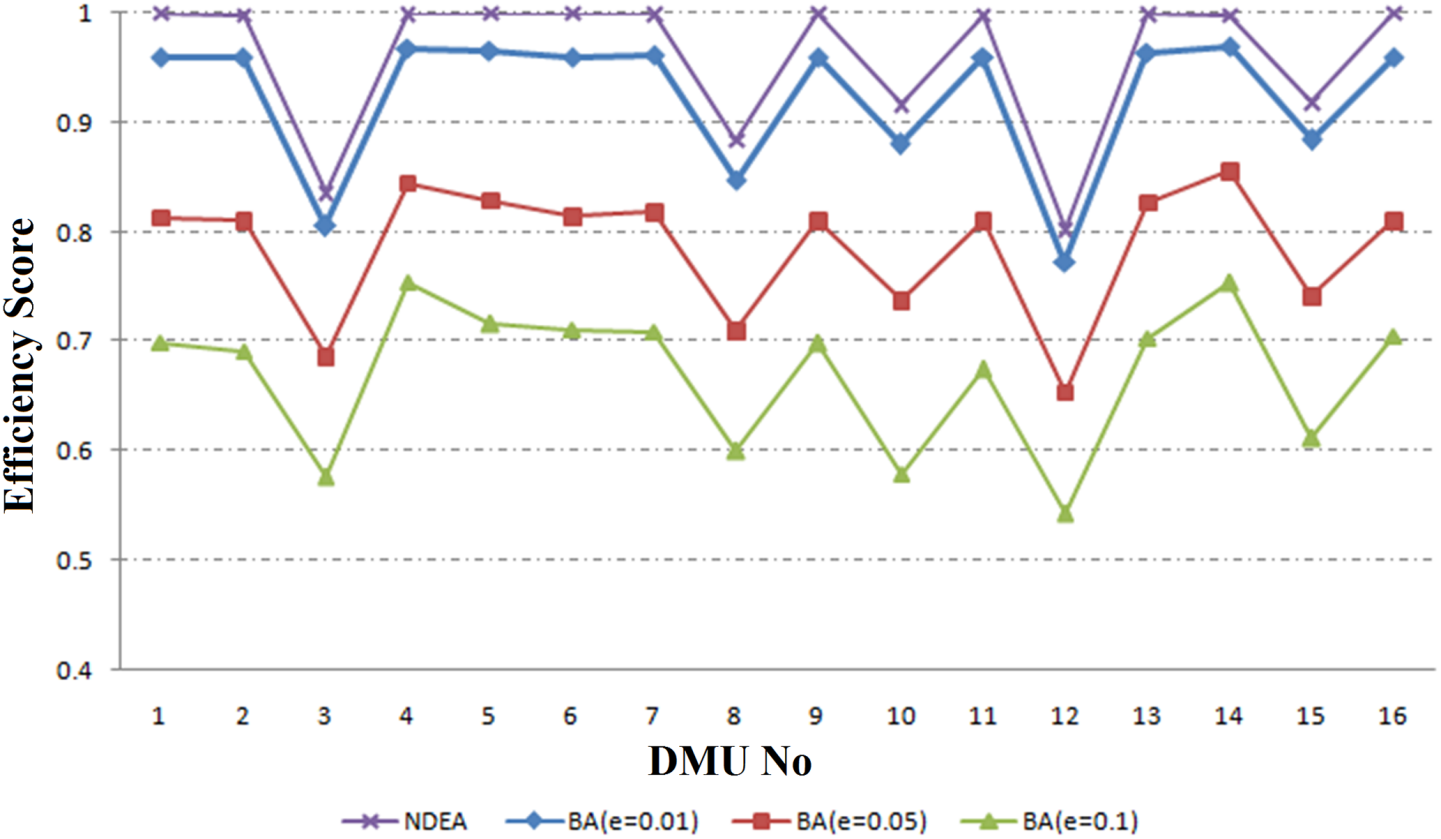
The results of RNDEA-BA approach.

Fig 4 and Table 2 (columns 9–11) show the results of RNDEA-BA approach. In this case, as perturbation increases from 0.01 to 0.1, the mean of efficiency measures is decreased from 0.924 to 0.670 and for each DMU, when the perturbation increases, the efficiency score decreases.

The process of implementing proposed framework is as follows: first managers collect data from 16 regional electricity power networks. Then, according to their knowledge about DMUs, they determine that data from which DMUs are subject to uncertainty. After that, they decide which method (RNDEA-BA or RNDEA-BN) should be applied (for DMUs with certain data they use conventional network DEA method). Then, for each DMU with uncertain data, they determine the uncertainty level. Finally, they use Table 2, or Figs 3 or 4, to measure the efficiency of DMUs and rank them.

Choosing between applying RNDEA-BN or RNDEA-BA approaches is a managerial decision and we cannot determine which one is more accurate. Note that if decision makers decide to use RNDEA-BN (or RNDEA-BA) approach, they should use it for all DMUs with uncertain data, i.e., it is not correct to use BN approach for some DMUs with uncertain data and use BA approach for other DMUs with uncertain data.

### SFA results

The SFA is applied as an alternative method to measure the efficiency of 16 electricity distribution networks. As our case is in multi-input and multi-output state, a translog distance function is used to estimate the parameters of SFA. The SFA method is implemented by using the FRONTIER 4.1 [62] to measure the efficiency of generation, transmission and distribution stages and then the efficiency of each network is average of efficiency scores in stages. The parameter *γ* for generation, transmission and distribution stages is 0.78, 0.99 and 0.82 respectively. The results of SFA method for 2014 are presented in third column of Table 2. As shown in this table, networks obtained efficiency scores between 0.776 and 0.996 and Fars and Azarbayejan are the least and the most efficient networks. The average of efficiency scores for 16 networks in 2014 is 0.927 and the standard deviation is 0.060.

### Comparison between BN and BA approaches

**Fig 5:**
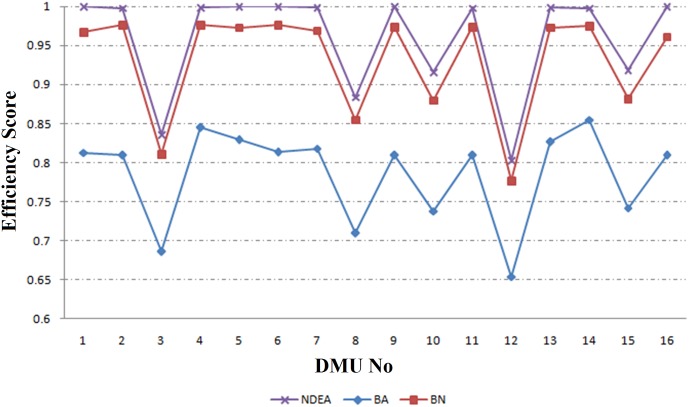
The results of network DEA, BA and BN approaches (e = 0.05).

The results of the Network DEA, BA and BN approaches are compared in Fig 5. The figure shows that efficiency scores in Robust Network DEA models are less that Network DEA. Also, efficiency scores of DMUs in BN method are less that BA method. While BN approach changes the class of problems, the BA approach preserves the class of problems, e.g., in BA approach the robust form of a linear programming model remains in linear programming form. However BN approach changes a linear programming model to a nonlinear one. Hence, if the number of constraints and variables increase, the BA approach is better than BN. In this study proposed robust network DEA models are in nonlinear form but the model based on BN approach is more complicated to solve.

Another indicators to compare BA and BN approaches, are the number of constrains and variables. Assume that there are *k* coefficients for the matrix *A* with *m* × *n* dimensions which are subject to uncertainty. Given that the original model has *n* variables and *m* constraints, BN approach has *m+2k* constraints and *n+2k* variables where *k* is the number of uncertain data. The BA approach has *m+k+n* constraints and *n+k+1* variables [6]. Therefore, BA has fewer variables than BN and when *k* > *n* the number of constraints in BA approach is less that BN.

### Comparison between SFA and robust network DEA approaches

**Fig 6:**
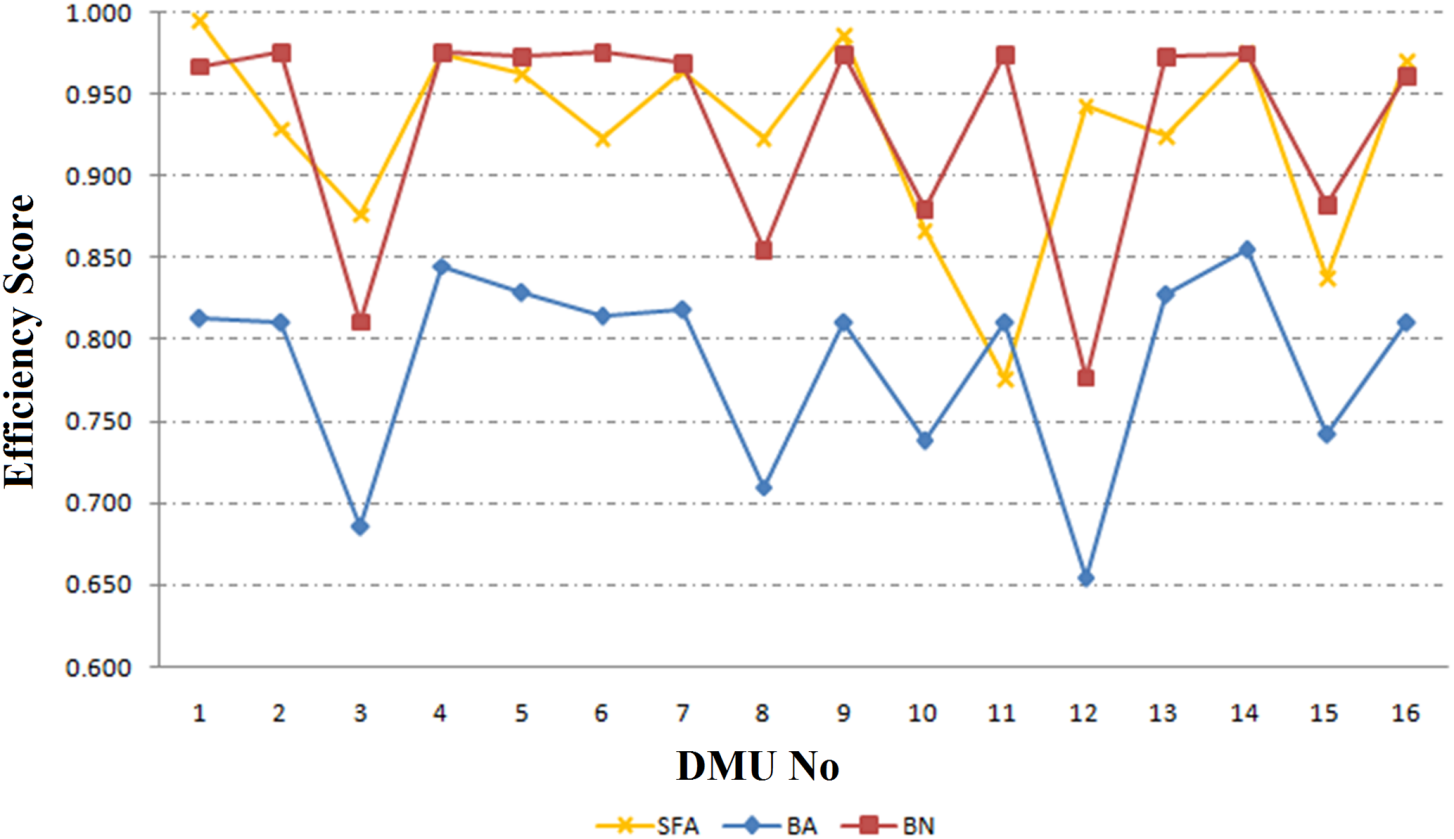
The results of SFA, BA and BN approaches (e = 0.05).

Fig 6 shows the results of SFA, BN and BA approaches. Clearly, the results of SFA are somewhat the same as BN or BA approaches. SFA method applies logarithmic equation and BN approach changes the class of problem and increases the number of variables. Hence, computationallythe BA approach performs better than SFA and BN approaches.

### Validation

To verify the results of proposed models, the Pearson test of correlation (*ρ*) and the Spearman test of correlation (*r*_*s*_) are employed. Correlation coefficient describes both the strength and the direction of the relationship between two variables and can range in value from −1 to +1, where 1 is total positive correlation, −1 is total negative correlation and 0 is no correlation. Pearson correlation coefficient is a measure of the linear correlation between two variables. The Spearman correlation coefficient is often used to evaluate relationship between ranked variables rather than the raw data. In this study, the Pearson test is applied to compare proposed models with network DEA model and Spearman test is employed to compare proposed models with results of SFA method. Table 3 represents the Pearson and Spearman test between proposed models and NDEA and SFA models.

**Table 3:** The correlarion coefficient between different models.

	RNDEA-BN approach (e = 0.1)	RNDEA-BA approach (e = 0.1)
NDEA	*ρ* = 0.871*	*ρ* = 0.923*
SFA	*r*_*s*_ = 0.576**	*r*_*s*_ = 0.554**

* Significant at the 1% level,

**significant at the 5% level.

The Pearson test statistics is 0.871 and 0.923 for RNDEA-BN and RNDEA-BA approaches, respectively. The result indicates a strong direct relationship between NDEA and proposed models based on BN and BA approaches which results in the rejection of *H*_*0*_ at 1% level. To measure the Spearman correlation coefficient first regional electricity power networks should be ranked based on their efficiency scores. The *r*_*s*_ for RNDEA-BN and RNDEA-BA approaches is 0.576 and 0.554, respectively that shows a relatively strong direct relationship between SFA and proposed models. The results are significant at the 5% level.

## Conclusion

In real-world problems, sometimes data are imprecise or vague. This study has conducted a framework to evaluate the efficiency of DMUs with network structure under uncertainty when the distribution of uncertain parameters is unknown. The proposed approach is based on the recently developed robust optimization approaches presented by Ben-Tal and Nemirovski [4] and Bertsimas et al. [6]. In this paper robust network DEA models were developed which can handle uncertainty of inputs, outputs and intermediate measures. While in literature the efficiency of components of electricity power networks was evaluated, in this paper the efficiency of the whole network was measured. Developed models were verified and validated by Pearson and Spearman correlation techniques.

Robust network DEA models developed in this paper was applied to evaluate the efficiency of 16 regional electricity power networks. The Pearson test was applied to compare the proposed models with network DEA model and the Spearman test was employed to compare the proposed models with the results of the SFA method. The results of Pearson and Spearman tests were significant at 1% level 5% level, respectively; hence, there was a direct relationship between proposed models and network DEA and SFA methods.

The results show that the Robust Network DEA models are more reliable than Network DEA model.

## Supporting information

S1 File Zip file containing Iranian electricity power industry statistics over 2013–2015. (ZIP)

## References

[1] Charnes A, Cooper WW, Rhodes E. Measuring the efficiency of decision making units. European journal of operational research. 1978;2(6):429–44.

[2] Cron WL, Sobol MG. The relationship between computerization and performance: a strategy for maximizing the economic benefits of computerization. Information & Management. 1983;6(3):171–81.

[3] Wang CH, Gopal RD, Zionts S. Use of data envelopment analysis in assessing information technology impact on firm performance. Annals of Operations Research. 1997;73:191–213.

[4] Ben-Tal A, Nemirovski A. Robust solutions of linear programming problems contaminated with uncertain data. Mathematical programming. 2000;88(3):411–24.

[5] Cooper WW, Seiford LM, Tone K. Data envelopment analysis: a comprehensive text with models, applications, references and DEA-solver software. Springer Science & Business Media; 2007.

[6] Bertsimas D, Pachamanova D, Sim M. Robust linear optimization under general norms. Operations Research Letters. 2004;32(6):510–6.

[7] Hu X, Martinez CM, Yang Y. Charging, power management, and battery degradation mitigation in plug-in hybrid electric vehicles: A unified cost-optimal approach. Mechanical Systems and Signal Processing. 2017;87:4–16.

[8] Hu X, Moura SJ, Murgovski N, Egardt B, Cao D. Integrated optimization of battery sizing, charging, and power management in plug-in hybrid electric vehicles. IEEE Transactions on Control Systems Technology. 2016;24(3):1036–43.

[9] Hu X, Zou Y, Yang Y. Greener plug-in hybrid electric vehicles incorporating renewable energy and rapid system optimization. Energy. 2016;111:971–80.

[10] Hu X, Jiang J, Egardt B, Cao D. Advanced power-source integration in hybrid electric vehicles: Multicriteria optimization approach. IEEE Transactions on Industrial Electronics. 2015;62(12):7847–58.

[11] Edvardsen DF, Førsund FR. International benchmarking of electricity distribution utilities. Resource and energy Economics. 2003;25(4):353–71.

[12] Estache A, Rossi MA, Ruzzier CA. The case for international coordination of electricity regulation: evidence from the measurement of efficiency in South America. Journal of Regulatory Economics. 2004;25(3):271–95.

[13] Giannakis D, Jamasb T, Pollitt M. Benchmarking and incentive regulation of quality of service: an application to the UK electricity distribution networks. Energy Policy. 2005;33(17):2256–71.

[14] Ramos-Real FJ, Tovar B, Iootty M, de Almeida EF, Pinto HQ. The evolution and main determinants of productivity in Brazilian electricity distribution 1998–2005: An empirical analysis. Energy Economics. 2009;31(2):298–305.

[15] Sadjadi SJ, Omrani H. Data envelopment analysis with uncertain data: An application for Iranian electricity distribution companies. Energy Policy. 2008;36(11):4247–54.

[16] Tavana M, Khakbaz MH, Jafari-Songhori M. Information technology's impact on productivity in conventional power plants. International Journal of Business Performance Management. 2009;11(3):187–202.

[17] Sözen A, Alp İ, Özdemirc A. Assessment of operational and environmental performance of the thermal power plants in Turkey by using data envelopment analysis. Energy Policy. 2010;38(10):6194–203.

[18] Vazhayil JP, Balasubramanian R. Optimization of India's power sector strategies using weight-restricted stochastic data envelopment analysis. Energy Policy. 2013;56:456–65.

[19] Charnes A, Cooper WW, Golany B, Halek R, Klopp G, Schmitz E, et al. Two phase data envelopment analysis approaches to policy evaluation and management of army recruiting activities: Tradeoffs between joint services and army advertising: Research report CCS; 1986 Contract No.: Document Number|.

[20] Färe R, Grosskopf S, Brännlund R. Intertemporal production frontiers: with dynamic DEA. Kluwer Academic Boston; 1996.

[21] Prieto AM, Zofío JL. Network DEA efficiency in input-output models: With an application to OECD countries. European journal of operational research. 2007;178(1):292–304.

[22] Cook WD, Zhu J, Bi G, Yang F. Network DEA: Additive efficiency decomposition. European journal of operational research. 2010;207(2):1122–9.

[23] Kao C. Efficiency decomposition in network data envelopment analysis: A relational model. European journal of operational research. 2009;192(3):949–62.

[24] Saranga H, Moser R. Performance evaluation of purchasing and supply management using value chain DEA approach. European journal of operational research. 2010;207(1):197–205.

[25] Yu M-M, Ting S-C, Chen M-C. Evaluating the cross-efficiency of information sharing in supply chains. Expert Systems with Applications. 2010;37(4):2891–7.

[26] Chen C, Yan H. Network DEA model for supply chain performance evaluation. European journal of operational research. 2011;213(1):147–55.

[27] Kao C. Network data envelopment analysis: A review. European journal of operational research. 2014;239(1):1–16. 10.1016/j.ejor.2020.09.044 PMC7534632 33041472

[28] Kao C. Interval efficiency measures in data envelopment analysis with imprecise data. European journal of operational research. 2006;174(2):1087–99.

[29] Wang Y-M, Greatbanks R, Yang J-B. Interval efficiency assessment using data envelopment analysis. Fuzzy sets and Systems. 2005;153(3):347–70.

[30] Lertworasirikul S, Fang S-C, Joines JA, Nuttle HLW. Fuzzy data envelopment analysis (DEA): a possibility approach. Fuzzy sets and Systems. 2003;139(2):379–94.

[31] Saati SM, Memariani A, Jahanshahloo GR. Efficiency analysis and ranking of DMUs with fuzzy data. Fuzzy Optimization and Decision Making. 2002;1(3):255–67.

[32] Sengupta JK. A fuzzy systems approach in data envelopment analysis. Computers & Mathematics with Applications. 1992;24(8):259–66.

[33] Cooper WW, Park KS, Yu G. IDEA and AR-IDEA: Models for dealing with imprecise data in DEA. management science. 1999;45(4):597–607.

[34] Soleimani-Damaneh M, Jahanshahloo GR, Abbasbandy S. Computational and theoretical pitfalls in some current performance measurement techniques; and a new approach. Applied mathematics and computation. 2006;181(2):1199–207.

[35] Soyster AL. convex programming with set-inclusive constraints and applications to inexact linear programming. Operations research. 1973;21(5):1154–7.

[36] El-Ghaoui L, Lebret H. Robust solutions to least-square problems to uncertain data matrices. Sima Journal on Matrix Analysis and Applications. 1997;18:1035–64.

[37] El Ghaoui L, Oustry F, Lebret H. Robust solutions to uncertain semidefinite programs. SIAM Journal on Optimization. 1998;9(1):33–52.

[38] Ben-Tal A, Nemirovski A. Robust convex optimization. Mathematics of Operations Research. 1998;23(4):769–805.

[39] Ben-Tal A, Nemirovski A. Robust solutions of uncertain linear programs. Operations Research Letters. 1999;25(1):1–13.

[40] Bertsimas D, Sim M. Robust discrete optimization and network flows. Mathematical programming. 2003;98(1):49–71.

[41] Bertsimas D, Sim M. The price of robustness. Operations research. 2004;52(1):35–53.

[42] Bertsimas D, Sim M. Tractable approximations to robust conic optimization problems. Mathematical programming. 2006;107(1–2):5–36.

[43] Cohen I, Golany B, Shtub A. The stochastic time-cost tradeoff problem: a robust optimization approach. Networks. 2007;49(2):175–88.

[44] Adida E, Joshi P. A robust optimisation approach to project scheduling and resource allocation. International Journal of Services Operations and Informatics. 2009;4(2):169–93.

[45] Wiesemann W, Kuhn D, Rustem B. Robust resource allocations in temporal networks. Mathematical programming. 2010;135(1–2):437–71.

[46] Bertsimas D, Thiele A. A robust optimization approach to inventory theory. Operations research. 2006;54(1):150–68.

[47] See C-T, Sim M. Robust approximation to multiperiod inventory management. Operations research. 2010;58(3):583–94.

[48] El Ghaoui L, Oks M, Oustry F. Worst-case value-at-risk and robust portfolio optimization: A conic programming approach. Operations research. 2003;51(4):543–56.

[49] Fabozzi FJ, Kolm PN, Pachamanova D, Focardi SM. Robust portfolio optimization and management. John Wiley & Sons; 2007.

[50] Bertsimas D, Pachamanova D. Robust multiperiod portfolio management in the presence of transaction costs. Computers & Operations Research. 2008;35(1):3–17.

[51] Nie XH, Huang GH, Li YP, Liu L. IFRP: A hybrid interval-parameter fuzzy robust programming approach for waste management planning under uncertainty. Journal of environmental management. 2007;84(1):1–11. doi: 10.1016/j.jenvman.2006.04.006 16854517 10.1016/j.jenvman.2006.04.006

[52] Li YP, Huang GH, Nie XH, Nie SL. A two-stage fuzzy robust integer programming approach for capacity planning of environmental management systems. European journal of operational research. 2008;189(2):399–420.

[53] Ben-Tal A, El Ghaoui L, Nemirovski A. Robust optimization. Princeton University Press; 2009.

[54] Beyer H-G, Sendhoff B. Robust optimization-a comprehensive survey. Computer methods in applied mechanics and engineering. 2007;196(33):3190–218.

[55] Kao C, Hwang S-N. Efficiency decomposition in two-stage data envelopment analysis: An application to non-life insurance companies in Taiwan. European journal of operational research. 2008;185(1):418–29.

[56] Aigner D, Lovell CAK, Schmidt P. Formulation and estimation of stochastic frontier production function models. journal of Econometrics. 1977;6(1):21–37.

[57] Meeusen W, Van den Broeck J. Efficiency estimation from Cobb-Douglas production functions with composed error. International economic review. 1977:435–44.

[58] Jamasb T, Pollitt M. International benchmarking and regulation: an application to European electricity distribution utilities. Energy Policy. 2003;31(15):1609–22.

[59] Kumbhakar SC, Lovell CAK. Stochastic frontier analysis. Cambridge University Press; 2003.

[60] Färe R, Grosskopf S, Lovell CAK, Yaisawarng S. Derivation of shadow prices for undesirable outputs: a distance function approach. The review of economics and statistics. 1993:374–80.

[61] Herrero I. Different approaches to efficiency analysis. An application to the Spanish Trawl fleet operating in Moroccan waters. European journal of operational research. 2005;167(1):257–71.

[62] Coelli TJ. A guide to FRONTIER version 4.1: a computer program for stochastic frontier production and cost function estimation: CEPA working paper; 1996 Contract No.: Document Number|.

